# Antimicrobial resistance among *Enterobacteriaceae*, *Staphylococcus aureus*, and *Pseudomonas* spp. isolates from clinical specimens from a hospital in Nairobi, Kenya

**DOI:** 10.7717/peerj.11958

**Published:** 2021-09-01

**Authors:** Jennifer Lord, Anthony Gikonyo, Amos Miwa, Agricola Odoi

**Affiliations:** 1 Biomedical and Diagnostic Sciences, University of Tennessee, Knoxville, TN, United States of America; 2 The Karen Hospital, Nairobi, Kenya

**Keywords:** Epidemiologic study, Antimicrobial resistance, Multi-drug resistance, Firth logistic regression model, *Staphylococcus aureus*, *Enterobacteriaceae*, *Pseudomonas*, Kenya

## Abstract

**Background:**

Antimicrobial resistance among pathogens of public health importance is an emerging problem in sub-Saharan Africa. Unfortunately, published information on the burden and patterns of antimicrobial resistance (AMR) in this region is sparse. There is evidence that the burden and patterns of AMR vary by geography and facility. Knowledge of local epidemiology of AMR is thus important for guiding clinical decisions and mitigation strategies. Therefore, the objective of this study was to determine the burden and predictors of AMR and multidrug resistance (MDR) among bacterial pathogens isolated from specimens submitted to the diagnostic laboratory of a hospital in Nairobi, Kenya.

**Methods:**

This retrospective study used laboratory records of 1,217 clinical specimens submitted for bacterial culture and sensitivity testing at the diagnostic laboratory of The Karen Hospital in Nairobi, Kenya between 2012 and 2016. Records from specimens positive for *Enterobacteriaceae*, *Staphylococcus aureus*, or *Pseudomonas* spp. isolates were included for analysis. Firth logistic models, which minimize small sample bias, were used to investigate determinants of AMR and MDR of the isolates.

**Results:**

A total of 222 specimens had bacterial growth. Most *Enterobacteriaceae* isolates were resistant to commonly used drugs such as penicillin/β-lactamase inhibitor combinations (91.2%) and folate pathway inhibitors (83.7%). Resistance to extended-spectrum cephalosporins was also high (52.9%). Levels of AMR and MDR for *Enterobacteriaceae* were 88.5% and 51%, respectively. Among *S. aureus* isolates, 57.1% were AMR, while 16.7% were MDR. As many as 42.1% of the *Pseudomonas* spp. isolates were aminoglycoside-resistant and 15% were fluoroquinolone-resistant, but none exhibited resistance to antipseudomonal carbapenems. Half of *Pseudomonas* spp. isolates were AMR but none were MDR. Significant predictors of MDR among *Enterobacteriaceae* were organism species (*p* = 0.002) and patient gender (*p* = 0.024).

**Conclusions:**

The high levels of extended-spectrum cephalosporin resistance and MDR among *Enterobacteriaceae* isolates are concerning. However, the relatively low levels of MDR *S. aureus,* and an absence of carbapenem resistance among *Pseudomonas* isolates, suggests that last-line drugs are still effective against *S. aureus* and *Pseudomonas* infections. These findings are relevant for guiding evidence-based treatment decisions as well as surveillance efforts and directions for future research, and contribute to the sparse literature on AMR in sub-Saharan Africa.

## Background

The development and spread of antimicrobial resistance (AMR) is a barrier to the effective treatment of infectious diseases, and contributes to adverse health outcomes including treatment failure, prolonged illness, and mortality (World Health Organization, 2014). The economic cost of the problem is predicted to be higher in low- and middle-income countries than the developed economies (World Bank Group, 2017). In Kenya, drug-resistant bacteria have been implicated in both nosocomial and community-acquired infections (Kariuki et al., 2003; Kariuki et al., 2006; Kariuki et al., 2007; Huber et al., 2014; Henson et al., 2017). Moreover, infections with pathogens included in the World Health Organization’s (WHO’s) priority list (e.g., MDR *Enterobacteriaceae,* methicillin-resistant *Staphylococcus aureus* (MRSA), and carbapenem-resistant *Pseudomonas aeruginosa* and *Acinetobacter baumannii*) have been reported in the country (Pitout et al., 2008; Huber et al., 2014; World Health Organization, 2017; Musyoki et al., 2019; Thuo et al., 2019; Wangai et al., 2019).

*Enterobacteriaceae* that are resistant to third-generation cephalosporins and carbapenems have been designated as critically important for public health because there are few remaining treatment options for these infections (World Health Organization, 2017). Recent evidence from Kenya suggests that resistance to extended-spectrum cephalosporins is an emerging problem in the country (Apondi et al., 2016; Taitt et al., 2017; Henson et al., 2017; Wangai et al., 2019).

Methicillin-resistant *S. aureus,* another priority pathogen, is well known for its role in both community- and healthcare-associated infections worldwide (Stefani et al., 2012; Mediavilla et al., 2012). Both MRSA and vancomycin-resistant *S. aureus* have been detected in environmental samples from a Kenyan teaching hospital (Muriungi Mbogori & Kiilu Musyoki, 2017). Moreover, some studies have reported high proportions of MRSA in Kenya (Maina et al., 2013a; Wangai et al., 2019).

Multidrug-resistant *Pseudomonas aeruginosa* is another cause of potentially life-threatening infections, and carbapenem-resistant isolates have been designated as “critical” on the WHO priority pathogens list (Pitout et al., 2008; World Health Organization, 2017). One of the first reports of healthcare-associated infections with carbapenem-resistant *P. aeruginosa* in Africa was in 2005, from a teaching hospital in Kenya where 13.7% of hospital *P. aeruginosa* isolates were resistant to both imipenem and meropenem (Pitout et al., 2008). Hospitals in Kenya have reported even higher levels of meropenem resistance among *P. aeruginosa* isolates (Mukaya et al., 2018; Wangai et al., 2019), suggesting that carbapenem resistance is an emerging problem.

The above reports are concerning and hence epidemiologic surveillance, with an emphasis on pathogens of high priority for public health, is warranted to gain a more comprehensive understanding of the burden of AMR and MDR. Encouragingly, Kenya is enrolled in the WHO Global Antimicrobial Surveillance System (GLASS), and is building a national AMR surveillance system (World Health Organization, 2018). However, the findings of locally focused studies are useful to complement these broader surveillance efforts (Kariuki et al., 2018). Information from such local studies are useful for guiding clinical decisions and evidence-based control strategies. Therefore, the objective of this study was to determine the burden and predictors of antimicrobial and multidrug resistance among bacterial pathogens isolated from specimens submitted to the diagnostic laboratory of a hospital in Nairobi, Kenya.

## Methods

### Design, setting and data source

This retrospective epidemiologic study used laboratory records of 1,217 clinical specimens submitted to the diagnostic laboratory of The Karen Hospital for bacterial culture and sensitivity testing between 2012 and 2016. Records of specimens positive for *Enterobacteriaceae*, *Staphylococcus aureus*, or *Pseudomonas* spp. were included in the study. Only records of these organisms were included for further analysis because they were the most frequently isolated organisms and sample sizes of other organisms were not sufficient to enable meaningful analysis. The following data were extracted from the laboratory records of specimens selected for inclusion in the study: medical record number, sample identification, patient gender, age, specimen site, bacterial growth, bacterial species isolated, and results of antimicrobial susceptibility testing. Hospital records for both inpatient and outpatient visits were also obtained. The following data were extracted from the hospital records: medical record number, marital status, visit date, admission date, discharge date, and inpatient/outpatient designation.

### Bacterial isolation and antimicrobial susceptibility testing

The hospital laboratory followed the European Committee on Antimicrobial Susceptibility testing (EUCAST) procedures for bacterial isolation and antimicrobial susceptibility testing (The European Committee on Antimicrobial Susceptibility Testing, 2012a; The European Committee on Antimicrobial Susceptibility Testing, 2013a; The European Committee on Antimicrobial Susceptibility Testing, 2014a; The European Committee on Antimicrobial Susceptibility Testing, 2015a). Antimicrobial susceptibility testing was performed using the Kirby-Bauer disk diffusion method (The European Committee on Antimicrobial Susceptibility Testing, 2012a; The European Committee on Antimicrobial Susceptibility Testing, 2013a; The European Committee on Antimicrobial Susceptibility Testing, 2014a; The European Committee on Antimicrobial Susceptibility Testing, 2015a). Isolates were classified as susceptible, intermediate or resistant based on breakpoints listed in the EUCAST guidelines (The European Committee on Antimicrobial Susceptibility Testing, 2012b; The European Committee on Antimicrobial Susceptibility Testing, 2013b; The European Committee on Antimicrobial Susceptibility Testing, 2014b; The European Committee on Antimicrobial Susceptibility Testing, 2015b; The European Committee on Antimicrobial Susceptibility Testing, 2016a). The laboratory followed EUCAST guidelines for routine quality control, using EUCAST-recommended quality control strains (The European Committee on Antimicrobial Susceptibility Testing, 2012c; The European Committee on Antimicrobial Susceptibility Testing, 2012d; The European Committee on Antimicrobial Susceptibility Testing, 2013c; The European Committee on Antimicrobial Susceptibility Testing, 2014c; The European Committee on Antimicrobial Susceptibility Testing, 2015c; The European Committee on Antimicrobial Susceptibility Testing, 2016b).

### Data management

Data management was performed in SAS 9.4 (SAS Institute, Cary, NC, USA). The data were assessed for duplicate entries but none were found. Patient hospital records were joined to laboratory records based on medical record numbers and if the date of hospital visit, admission, or discharge occurred within 14 days of sample submission or laboratory report. Patient entries were coded as “inpatient” or “outpatient” based on a visit identification indicator when available. If a visit identification indicator was not available, hospital status (inpatient *vs* outpatient) was determined based upon the presence or absence of an admission date.

Records for non-duplicate specimens positive for *Enterobacteriaceae*, *Staphylococcus aureus*, or *Pseudomonas* spp. isolates were included for further analysis in the study because these were the most frequently isolated organisms. Specimen site was re-categorized to reflect the most common sites of sample collection. For enteric bacteria and *Pseudomonas* spp. this included stool, blood, and sputum. Isolates obtained from all other sites were categorized as “other”. The most common specimen sites for *S. aureus* isolates were blood, wounds and abscesses; isolates from all other sites were categorized as “other”.

The results of susceptibility testing were re-coded to two categories, “resistant” and “susceptible”. Isolates with “intermediate” susceptibility were re-coded as resistant (Magiorakos et al., 2012). In order to further classify isolates as antimicrobial or multidrug resistant, antimicrobial agents were placed into clinically significant antimicrobial categories listed in guidelines established by the European Center for Disease Prevention and Control (ECDC) and United States Centers for Disease Control and Prevention (CDC) (Magiorakos et al., 2012). Isolates resistant to one or more drugs in at least one category were considered to be antimicrobial resistant (AMR). Isolates resistant to one or more antimicrobial agents in at least three categories were classified as multidrug resistant (MDR) (Magiorakos et al., 2012). If a bacterial species was intrinsically resistant to an antimicrobial agent or category, results of susceptibility testing for isolates of that species were not reported, and the antimicrobial was excluded during classification of the isolate as AMR or MDR (Bouza & Cercenado, 2002; Pfeifer, Cullik & Witte, 2010; Magiorakos et al., 2012; Leclercq et al., 2013; Ruppé, Woerther & Barbier, 2015; Hughes et al., 2016; Goto et al., 2017; CLSI, 2020).

The following antimicrobial categories and agents were used in the classification of *Enterobacteriaceae*: aminoglycosides (gentamicin, amikacin, kanamycin, streptomycin), carbapenems (ertapenem, imipenem, imipenem/cilastatin, doripenem, meropenem), non-extended spectrum cephalosporins (cefuroxime, cefaclor, cephalexin), extended-spectrum cephalosporins (ceftriaxone, ceftazidime), quinolones and fluoroquinolones (ciprofloxacin, nalidixic acid, norfloxacin, ofloxacin), folate pathway inhibitors (sulfamethoxazole +/- trimethoprim), penicillins (ampicillin, cloxacillin, methicillin, piperacillin), penicillin/β-lactamase inhibitor combinations (amoxicillin/clavulanate), phenicols (chloramphenicol), and tetracyclines (tetracycline, minocycline).

Antimicrobial categories and agents used in the classification of *S. aureus* isolates included: aminoglycosides (gentamicin, amikacin, kanamycin, streptomycin), anti-staphylococcal β-lactams (oxacillin, methicillin, cloxacillin, flucloxacillin), fluoroquinolones (ciprofloxacin, norfloxacin, ofloxacin), folate pathway inhibitors (sulfamethoxazole +/- trimethoprim), glycopeptides (vancomycin), lincosamides (lincomycin), macrolides (erythromycin), phenicols (chloramphenicol), and tetracyclines (tetracycline, minocycline). For *Pseudomonas* spp. isolates, the following categories and agents were used: aminoglycosides (gentamicin, amikacin), antipseudomonal carbapenems (meropenem, doripenem), and antipseudomonal fluoroquinolones (ciprofloxacin, ofloxacin).

### Statistical analysis

All statistical analyses were performed using SAS 9.4 (SAS Institute, Cary, NC, USA). The Shapiro–Wilk test was used to assess for normality of distribution of patient age. Since patient age was non-normally distributed, median and interquartile range were used as measures of central tendency and dispersion. The Cochran-Armitage trend test was used to assess for significant temporal trends in AMR and MDR. Univariable Firth logistic regression models were used to assess whether resistance to one antimicrobial class could be used to predict resistance to other antimicrobial classes among *Enterobacteriaceae* isolates. The Firth logistic regression models were necessary for these analyses because of the small number of isolates in some of the analyses. Ordinary logistic models, which use maximum likelihood estimation, are not appropriate for these models because they suffer from small-sample bias (Heinze & Schemper, 2002). To mitigate small-sample bias, the Firth models use penalized likelihood functions to obtain parameter estimates rather than maximum likelihood used by ordinary logistic models (Firth, 1993).

The following potential explanatory variables were investigated for associations with AMR and MDR: patient hospitalization status (inpatient *vs* outpatient), specimen site, patient gender, patient age category, patient marital status, and bacterial species isolated (for *Enterobacteriaceae*). Firth logistic regression models were used to investigate determinants of: (a) AMR and MDR among enteric bacteria, (b) AMR and MDR among *S. aureus* isolates, and (c) AMR among *Pseudomonas* spp. isolates. Model building was performed using a two-step process. First, univariable Firth logistic regression models were used to identify explanatory variables significantly associated with each of the outcomes. In the second step, variables that had significant univariable associations with the outcomes (using a relaxed *p*-value of <0.15) were considered in multivariable Firth logistic regression models. Model building was performed using manual backwards elimination, with a cutoff *p-* value of ≤0.05. Explanatory variables were considered to be confounders if their removal resulted in >20% change in the coefficients of the variables in the model. Model fit was assessed using the Hosmer-Lemeshow goodness-of-fit test.

### Ethics approval

This study was approved by the Standards and Ethics Committee of The Karen Hospital and the University of Tennessee Institutional Review Board. UTK IRB16-03358-XM. The clinical specimens used in this study were collected as part of the routine diagnostic testing procedures used for patient care in the hospital. Since this was a retrospective study, no contact was made with patients and no attempt was made to link records to specific patients.

## Results

### Descriptive statistics

Of the 1,217 specimens submitted for culture and sensitivity testing, 222 had bacterial growth of which 166 met the inclusion criteria and were included for further analysis. The most frequently isolated pathogens were enteric bacteria (108 specimens), followed by *S. aureus* (42), and *Pseudomonas* spp. (20). Other bacterial species isolated included: *Acinetobacter* spp., *Enterococcus* spp., *Helicobacter* spp., *Moraxella catarrhalis,* coagulase negative *Staphylococcus* spp., untyped *Staphylococcus* spp., *Streptococcus pneumoniae, Streptococcus viridans,* and untyped *Streptococcus* spp.

Among the *Enterobacteriaceae*, *Klebsiella* spp. were the most frequently isolated (28.7%), followed by *Escherichia coli* (26.9%), *Proteus* spp. (15.7%), and *Salmonella* spp. (14.8%) (Table 1). Bacterial species included in the “other” category were: *Citrobacter* spp., *Enterobacter* spp., *Raoultella ornithinolytica,* and *Shigella* spp. Most (64.4%) enteric bacterial isolates were from inpatients, and approximately half (51.1%) were obtained from stool specimens (Tables 1 and 2). The majority of specimens positive for *Enterobacteriaceae* were collected in 2015 (42) and 2016 (48). There was an approximately even gender distribution, with 47.2% of *Enterobacteriaceae* isolated from female patients and 52.8% from male patients. Age of patients from whom enteric bacteria were isolated ranged from 1 to 93 years, with a median of 46.5 years and an interquartile range of 23–70.

**Table 1:** Patient and sample characteristics of bacterial isolates from specimens submitted to a diagnostic laboratory in Nairobi, Kenya (2012–2016).

**Variable**	**Classification**	***Enterobacteriaceae***	***S. aureus***	***Pseudomonas***
		Percent (frequency)	Percent (frequency)	Percent (frequency)
**Organism**	** **	** **	–	–
	*Escherichia coli*	26.9 (29/108)		
	*Proteus* spp.	15.7 (17/108)		
	*Klebsiella* spp.	28.7 (31/108)		
	*Salmonella* spp.	14.8 (16/108)		
** **	Other or unspecified	13.9 (15/108)		
**Hospitalization status**	** **	** **		
	Inpatient	64.4 (29/45)	77.3 (17/22)	85.7 (6/7)
** **	Outpatient	35.6 (16/45)	22.7 (5/22)	14.3 (1/7)
Specimen site^*^	** **	** **		
	Stool	51.1 (48/94)	–	18.2 (2/11)
	Sputum	13.8 (13/94)	–	45.5 (5/11)
	Blood	13.8 (13/94)	48.3 (14/29)	18.2 (2/11)
	Wound/abscess	–	31 (9/29)	–
** **	Other	21.3 (20/94)	20.7 (6/29)	18.2 (2/11)
**Gender**	** **	** **		
	Female	47.2 (51/108)	35.7 (15/42)	20 (4/20)
** **	Male	52.8 (57/108)	64.3 (27/42)	80 (16/20)
**Age category**	** **	** **		
	<25 years	25 (27/108)	21.4 (9/42)	10 (2/20)
	25–45 years	22.2 (24/108)	38.1 (16/42)	15 (3/20)
	45–65 years	25 (27/108)	21.4 (9/42)	15 (3/20)
** **	>65 years	27.8 (30/108)	19.1 (8/42)	60 (12/20)
**Marital status**	** **	** **		
	Married	56.6 (43/76)	67.7 (21/31)	64.3 (9/14)
	Single	29 (22/76)	22.6 (7/31)	14.3 (2/14)
** **	Other	14.5 (11/76)	9.7 (3/31)	21.4 (3/14)

**Notes.**

* Specimen site categories used for *Enterobacteriaceae* and *Pseudomonas* spp. isolates: stool, sputum, blood, all others. Specimen site categories used for *S. aureus* isolates: blood, wound/abscess, all others.

**Table 2:** Specimen site distribution of *Enterobacteriaceae* isolated from specimens submitted to a diagnostic laboratory in Nairobi, Kenya (2012–2016).

**Organism**	**Specimen site**				**Total**
	**Blood**	**Sputum**	**Stool**	**Other**	
*Citrobacter* spp.	2	0	2	0	4
*Enterobacter cloacae*	0	1	0	0	1
*Escherichia coli*	7	0	11	7	25
*Klebsiella* spp.	3	10	5	8	26
*Proteus* spp.	0	0	12	2	14
*Raoultella ornithinolytica*	0	1	0	0	1
*Salmonella* spp.	0	0	15	0	15
*Shigella* spp.	0	0	1	0	1
Unspecified *Enterobacteriaceae*	1	1	2	3	7

The majority (77.3%) of *S. aureus* isolates were also from inpatients, and blood was the most common specimen (48.3%) followed by wound or abscess (31.0%) (Table 1). Specimen sites in the “other” category included sputum (four), eye swab (one), and central venous catheter (one). Most (64.3%) *S. aureus* positive samples were from males. Patient age for *S. aureus* positive samples ranged from <1 year to 88 years, with a mean of 40.8 years. The majority of patients with *S. aureus* infections were married (67.7%). As with *Enterobacteriaceae*, most *S. aureus* positive specimens were collected in 2015 (17) and 2016 (17).

Almost half (45.5%) of *Pseudomonas* spp. isolates were obtained from sputum samples, followed by blood (18.2%) and stool (18.2%). The majority of specimens positive for *Pseudomonas* spp. were from male (80.0%) and married (64.3%) patients. The age of patients who were positive for *Pseudomonas* spp. ranged from 21 to 85 years, with a median of 71 years and an interquartile range of 43–82.

### Patterns of antimicrobial and multidrug resistance

Overall, 75.9% of the isolates included in the current study were AMR, while 36.1% were MDR. There were no significant temporal trends in the overall burden of AMR (*p* = 0.2489) or MDR (*p* = 0.6340).

### (a) Enterobacteriaceae

Susceptibility test results for one or more antimicrobial agents were available for 104 of the 108 enteric bacterial isolates. Among *Enterobacteriaceae*, the percentages of resistant isolates were highest for the following antimicrobial categories: penicillin/β-lactamase inhibitor combinations (91.2%), folate pathway inhibitors (83.7%), and penicillins (67.6%) (Table 3). Resistance to extended-spectrum cephalosporins was observed in about half (52.9%) of *Enterobacteriaceae* isolates. In addition, about one-third (30%) of the isolates exhibited resistance to at least one quinolone and/or fluoroquinolone, including 25.3% (22/87) that were resistant to one or more fluoroquinolones. Enteric bacteria had the lowest levels of resistance (6.5%) to carbapenems. As many as 88.5% and 51% of the *Enterobacteriaceae* isolates were AMR and MDR, respectively (Table 4). Moreover, 6.7% of the isolates exhibited MDR involving five antimicrobial categories (Table 4). While AMR exhibited a statistically significant temporal decrease between 2013 and 2016 (*p* = 0.0016), no significant temporal trends were observed for MDR (*p* = 0.4258).

**Table 3:** Antimicrobial resistance among bacterial isolates from specimens submitted to a diagnostic laboratory in Nairobi, Kenya (2012–2016).

**Organism**	**Antimicrobial category**	**Percent**	**Frequency**
***Enterobacteriaceae***	Aminoglycosides	27.6	27/98
	Carbapenems	6.5	5/72
	Non-extended spectrum cephalosporins (1st & 2nd generation)	100	1/1
	Extended-spectrum cephalosporins (3rd & 4th generation)	52.9	37/70
	Quinolones & fluoroquinolones	30.0	27/90
	Folate pathway inhibitors	83.7	36/43
	Penicillins	67.6	48/71
	Penicillin/ *β*-lactamase inhibitor combinations	91.2	52/57
	Phenicols	55.6	5/9
	Tetracyclines	50.0	8/16
***S. aureus***	Aminoglycosides	11.1	2/18
	Anti-staphylococcal *β*-lactams	4.8	1/21
	Fluoroquinolones	25.0	2/8
	Folate pathway inhibitors	56.7	17/30
	Glycopeptides	10.0	1/10
	Lincosamides	18.2	2/11
	Macrolides	29.6	8/27
	Phenicols	18.8	3/16
	Tetracyclines	35.0	7/20
***Pseudomonas*** **spp.**	Aminoglycosides	42.1	8/19
	Antipseudomonal carbapenems	0	0/19
	Antipseudomonal fluoroquinolones	15.0	3/20

**Table 4:** Antimicrobial and multidrug resistance among bacterial isolates from specimens submitted to a diagnostic laboratory in Nairobi, Kenya (2012–2016).

**Classification**	***Enterobacteriaceae***	***S. aureus***	***Pseudomonas*** **spp.**
	**Percent (frequency)**	**Percent (frequency)**	**Percent (frequency)**
AMR^1^	88.5 (92/104)	57.1 (24/42)	50 (10/20)
2012	–	–	–
2013	100 (2/2)	75 (3/4)	–
2014	100 (12/12)	50 (2/4)	–
2015	97.6 (41/42)	47.1 (8/17)	46.2 (6/13)
2016	77.1 (37/48)	64.7 (11/17)	66.7 (4/6)
MDR^2^	51 (53/104)	16.7 (7/42)	0 (0/20)
2012	–	–	–
2013	100 (2/2)	25 (1/4)	–
2014	50 (6/12)	25 (1/4)	–
2015	52.4 (22/42)	11.8 (2/17)	–
2016	47.9 (23/48)	17.7 (3/17)	–
No. resistant drug categories			
0	11.5 (12/104)	42.9 (18/42)	50 (10/20)
1	18.3 (19/104)	31 (13/42)	45 (9/20)
2	19.2 (20/104)	9.5 (4/42)	5 (1/20)
3	30.8 (32/104)	14.3 (6/42)	–
4	13.5 (14/104)	2.4 (1/42)	–
5	6.7 (7/104)	–	–

**Notes.**

1 Antimicrobial resistant.

2 Multidrug resistant.

Among *Enterobacteriaceae*, resistance to aminoglycosides was a significant predictor of resistance to carbapenems (Odds Ratio [OR] = 10.9; 95% confidence interval [CI] 1.3, 89.3; *p* = 0.027). In addition, resistance of enteric bacteria to extended-spectrum cephalosporins was a significant predictor (OR = 3.9; 95% CI 1.3, 11.6; *p* = 0.016) of resistance to quinolones and fluoroquinolones, as was resistance to penicillins (OR = 6.5; 95% CI 1.1, 40.3; *p* = 0.044). Resistance to extended-spectrum cephalosporins was also a predictor (OR = 17.9; 95% CI 3.7, 87.3; *p* = 0.0004) of resistance to penicillins.

### (b) S. aureus

Approximately half (56.7%) of the *S. aureus* isolates were resistant to folate pathway inhibitors, while approximately one-third were resistant to tetracyclines (35%) and macrolides (29.5%) (Table 3). Among *S. aureus* isolates, 57.1% were AMR, while 16.7% were MDR (Table 4). There were no significant temporal trends in AMR (*p* = 0.9409) or MDR (*p* = 0.6819) among the *S. aureus* isolates.

### (c) *Pseudomonas* spp

Among *Pseudomonas* spp. isolates, 42.1% were resistant to one or more aminoglycosides, and 15% were resistant to a fluoroquinolone (Table 3). Fifty percent of the *Pseudomonas* spp. isolates were AMR, and none were MDR (Table 4). *Pseudomonas* spp. isolates were only observed in 2015 and 2016, and there were no statistically significant differences (Fisher’s exact *p* = 0.6285) in the proportions of AMR isolates between the two years.

### Distribution of AMR and MDR isolates

The distribution of patient and specimen characteristics among AMR and MDR isolates, as well as *p-* values for associations assessed using univariable Firth logistic regression models, are displayed in Table 5. While the majority of *Klebsiella* spp. (71%) and *E. coli* (58.6%) isolates were MDR, only 23.5% of *Proteus* spp. and 14.3% of *Salmonella* spp. isolates were MDR. Stool specimens had the lowest proportion of MDR *Enterobacteriaceae* (36.4%), while about half of the *Enterobacteriaceae* isolates from sputum samples (46.2%) were MDR. The highest percentage of MDR isolates among enteric bacteria were from blood samples (69.2%) and other sites (75.0%) (Table 5). Older patients had the highest percentage of MDR infections, with 73.3% of *Enterobacteriaceae* isolated from those over 65 years of age exhibiting multidrug resistance.

**Table 5:** Distribution and univariable associations of AMR^1^ and MDR^2^ bacterial isolates from specimens submitted to a diagnostic laboratory in Nairobi, Kenya (2012–2016).

		***Enterobacteriaceae***		***S. aureus***		***Pseudomonas*** **spp.**
**Variable**	**Classification**	**AMR** ^1^	**MDR** ^2^	**AMR** ^1^	**MDR** ^2^	**AMR** ^1^
		% (n/N)	% (n/N)	% (n/N)	% (n/N)	% (n/N)
**Organism**		** ***p* = 0.763	** ***p* = 0.005	–	–	–
	*Escherichia coli*	93.1 (27/29)	58.6 (17/29)			
	*Proteus* spp.	88.2 (15/17)	23.5 (4/17)			
	*Klebsiella* spp.	87.1 (27/31)	71 (22/31)			
	*Salmonella* spp.	78.6 (11/14)	14.3 (2/14)			
	Other or unspecified	92.3 (12/13)	61.5 (8/13)			
**Hospitalization status**		*p* = 0.640	*p* = 0.334	*p* = 0.270	*p* = 0.788	*p* = 0.491
	Inpatient	96.4 (27/28)	53.6 (15/28)	70.6 (12/17)	17.7 (3/17)	33.3 (2/6)
** **	Outpatient	93.8 (15/16)	37.5 (6/16)	40 (2/5)	20 (1/5)	100 (1/1)
**Specimen site** ^*^	** **	** ***p* = 0.251	** ***p* = 0.031	*p* = 0.977	*p* = 0.259	*p* = 0.638
	Stool	81.8 (36/44)	36.4 (16/44)	–	–	50 (1/2)
	Sputum	76.9 (10/13)	46.2 (6/13)	–	–	80 (4/5)
	Blood	100 (13/13)	69.2 (9/13)	50 (7/14)	7.1 (1/14)	50 (1/2)
	Wound/abscess	–	–	55.6 (5/9)	22.2 (2/9)	–
** **	Other	100 (20/20)	75 (15/20)	50 (3/6)	33.3 (2/6)	0 (0/2)
**Gender**		*p* = 0.059	*p* = 0.127	*p* = 0.801	*p* = 0.747	*p* = 0.367
	Female	81.6 (40/49)	42.9 (21/49)	60 (9/15)	13.3 (2/15)	25 (1/4)
	Male	94.6 (52/55)	58.2 (32/55)	55.6 (15/27)	18.5 (5/27)	56.3 (9/16)
** Age category**		** ***p* = 0.304	** ***p* = 0.032	*p* = 0.661	*p* = 0.894	*p* = 0.887
	<25 years	88 (22/25)	52 (13/25)	55.6 (5/9)	22.2 (2/9)	50 (1/2)
	25–45 years	77.3 (17/22)	36.4 (8/22)	50 (8/16)	12.5 (2/16)	33.3 (1/3)
	45–65 years	88.9 (24/27)	37 (10/27)	77.8 (7/9)	22.2 (2/9)	33.3 (1/3)
	>65 years	96.7 (29/30)	73.3 (22/30)	50 (4/8)	12.5 (1/8)	58.33 (7/12)
**Marital status**		*p* = 0.641	*p* = 0.534	*p* = 0.591	*p* = 0.651	*p* = 0.700
	Married	90.5 (38/42)	50 (21/42)	66.7 (14/21)	14.3 (3/21)	66.7 (6/9)
	Single	90.9 (20/22)	63.6 (14/22)	42.9 (3/7)	28.6 (2/7)	50 (1/2)
	Other	81.8 (9/11)	45.5 (5/11)	66.7 (2/3)	0 (0/3)	33.3 (1/3)

**Notes.**

1 Antimicrobial resistant.

2 Multidrug resistant.

* Specimen site categories used for *Enterobacteriaceae* and *Pseudomonas* spp. isolates: stool, sputum, blood, all others. Specimen site categories used for *S. aureus* isolates: blood, wound/abscess, all others.

### Determinants of MDR

Species of the infectious agent and gender of the patient were significant predictors of MDR (Table 6). Compared to *E. coli* isolates, *Salmonella* spp. (OR = 0.10, *p* =0.008) and *Proteus* spp. (OR = 0.15, *p* =0.010) isolates had significantly lower odds of being multidrug resistant. However, there was no difference in the odds of MDR between *E. coli* and *Klebsiella* spp. isolates. *Enterobacteriaceae* isolated from male patients had significantly higher odds (OR = 3.0, *p* =0.024) of being MDR than those from female patients. Results of the Hosmer-Lemeshow test showed no evidence of lack of model fit to the data (*p* = 0.994).

**Table 6:** Predictors of MDR^1^ among *Enterobacteriaceae* isolated from specimens submitted to a diagnostic laboratory in Nairobi, Kenya (2012–2016).

**Variable**	**Classification**	**OR** ^2^	**95% CI** ^3^	***p*** **-value**
**Organism**				0.002
	*Proteus* spp.	0.15	0.04, 0.64	0.010
	*Klebsiella* spp.	1.6	0.52, 4.7	0.431
	*Salmonella* spp.	0.10	0.02, 0.54	0.008
	Other or unspecified	0.79	0.19, 3.2	0.737
** **	*Escherichia coli*	Referent	–	–
**Gender**				
	Male	3.0	1.2, 7.6	0.024
** **	Female	Referent	–	–

**Notes.**

1 Multidrug resistance.

2 Odds ratio.

3 Confidence interval.

## Discussion

This study investigated the burden of antimicrobial and multidrug resistance among the most frequently identified bacterial species isolated from clinical specimens submitted to a diagnostic laboratory in Nairobi, Kenya. Additionally, predictors of multidrug resistance were investigated. The findings are relevant for clinicians and may be useful in guiding evidence-based treatment decisions. Moreover, information from this study adds to the body of knowledge on antimicrobial resistance in sub-Saharan Africa, where there is limited published information on the burden and patterns of AMR and MDR. Continued surveillance to better characterize the burden of AMR, with a focus on pathogens of public health importance, is crucial to address the problem.

### (a) *Enterobacteriaceae*

#### Patterns of antimicrobial and multidrug resistance

The high levels of *Enterobacteriaceae* resistant to commonly used drugs such as penicillin/β-lactamase inhibitor combinations (91.2%) and folate pathway inhibitors isolates (83.7%) were consistent with findings of other studies conducted in East Africa. For example, a study from Kenya reported that 74% of *E. coli* and 73% of *K. pneumoniae* isolates from clinical samples were resistant to amoxicillin/clavulanate, while levels of resistance to trimethoprim/sulfamethoxazole (TMS) were 93% and 90%, respectively (Wangai et al., 2019). Other studies conducted in Kenya have also reported high levels of resistance to TMS (Sang, Oundo & Schnabel, 2012; Swierczewski et al., 2013). High levels of resistance to amoxicillin/clavulanate (71.6%) and TMS (77%) have also been reported among *Enterobacteriaceae* isolated from clinical specimens in Ethiopia (Teklu et al., 2019). These findings are not surprising, as these medications are inexpensive, orally available, and frequently used, leading to widespread selection for resistance in many countries in Africa (Leopold et al., 2014; Tadesse et al., 2017).

The levels of resistance to aminoglycosides (27.6%) among *Enterobacteriaceae* isolates were comparable to levels of gentamicin resistance among *Enterobacteriaceae* reported by Kenyan (16–35%) (Bejon et al., 2005) and Ethiopian (31.6%) studies (Bitew & Tsige, 2020). However, susceptibility to aminoglycosides has been reported to vary between individual drugs. Another Ethiopian study reported that while 43.4% of *Enterobacteriaceae* were resistant to gentamicin, far fewer (13.8%) were resistant to amikacin (Teklu et al., 2019).

Resistance to one or more fluoroquinolones was also observed among a considerable proportion (25.3%) of *Enterobacteriaceae* isolates. While this is concerning, it is not entirely surprising because the availability of oral formulations of these drugs make them quite popular and hence more likely to be misused (Chattaway et al., 2016). Fluoroquinolone resistance, while previously uncommon, has expanded relatively recently in sub-Saharan Africa compared to other parts of the world (Chattaway et al., 2016). Some recent reports from the region have described high levels of fluoroquinolone resistance among *Enterobacteriaceae* isolates. For instance, a recent Kenyan study reported that 28% of *P. mirabilis*, 56% of *K. pneumoniae,* and 78% of *E. coli* isolates exhibited resistance to ciprofloxacin (Wangai et al., 2019). Among *K. pneumoniae* isolated from blood cultures of patients in another hospital in Kenya, 44.4% were resistant to ciprofloxacin (Apondi et al., 2016). Similar proportions of fluoroquinolone-resistant *Enterobacteriaceae* (45.9%, 46.3%) were documented in reports from Ethiopia (Teklu et al., 2019; Bitew & Tsige, 2020). The current study found comparatively lower (25.3%), albeit substantial, levels of resistance to fluoroquinolones. This difference may reflect numerous factors, including patient characteristics, antimicrobial use patterns, and bacterial species, highlighting the importance of relevant local data for characterizing the burden of AMR.

The level of resistance to extended spectrum cephalosporins (52.9%) among *Enterobacteriaceae* isolates is of particular public health significance (World Health Organization, 2017). Extended-spectrum β-lactamase (ESBL) production is typically responsible for resistance to third-generation cephalosporins among *Enterobacteriaceae,* and plasmid-mediated mechanisms may be spread quickly between isolates (Ruppé, Woerther & Barbier, 2015). The findings from the current study were consistent with those of two laboratory-based studies in Ethiopia, where resistance to extended-spectrum cephalosporins ranged from 47.5% to 51.6% in one report, and 60.3% to 62.2% in another (Teklu et al., 2019; Bitew & Tsige, 2020). In contrast, a study conducted in Uganda reported resistance to third generation cephalosporins among *Enterobacteriaceae* isolates to be considerably lower (27.5%) (Ampaire et al., 2015).

The proportion of ESBL-producing isolates can differ between species of enteric bacteria, as evidenced by a study from Tanzania where ESBL production ranged from 24.4% among *E. coli* to 63.7% among *K. pneumoniae* isolates (Mshana et al., 2009). Similarly, levels of resistance to third generation cephalosporins among *E. coli* isolates (66–75%) were surpassed by *K. pneumoniae* (82–83%) in a recent report from Kenya (Wangai et al., 2019). Although there is apparent variation by geographic location and bacterial species, the substantial level of cephalosporin resistance observed in the current study, along with findings of other recent reports, suggests that cephalosporin resistance is an emerging problem. Unfortunately, very few studies have investigated the temporal trends of ESBL-producing isolates in sub-Saharan Africa. However, a significant increase in the proportion of ESBL-producing *K. pneumoniae* isolates was documented in a hospital in Kenya (Henson et al., 2017). A study from Zimbabwe also reported a temporal increase in ESBL production among *Enterobacteriaceae*, supporting the need to prioritize this issue (Mhondoro et al., 2019). While a temporal decrease in AMR among *Enterobacteriaceae* was observed in the current study, the ability to draw meaningful conclusions from this finding is limited by small sample size during the earlier years of this study. In addition, sample size precluded the assessment of temporal trends for individual drugs or antimicrobial categories. Clearly, further investigation of such trends is warranted in future, larger scale studies.

While the overall percentage (52.9%) of *Enterobacteriaceae* that exhibited resistance to extended-spectrum cephalosporins was quite concerning, far fewer carbapenem-resistant *Enterobacteriaceae* (CRE) were identified (6.5%). The findings of the current study are comparable to those from Ethiopia and Uganda, where carbapenem resistance was reported in 5.2% and 3.5% of enteric isolates, although additional isolates with genetic determinants of carbapenem resistance were identified in the Ugandan study (Ampaire et al., 2015; Teklu et al., 2019). Even lower levels of CRE (<1%) have been documented in other reports from Kenya and Zimbabwe (Maina et al., 2013b; Mhondoro et al., 2019). In contrast, some hospitals within Kenya have reported high levels of carbapenem resistance, particularly for *K. pneumoniae,* which is a serious public health concern (Apondi et al., 2016; Wangai et al., 2019). Carbapenems are considered “last-line” antimicrobials, and a very limited number of treatment options exist for infections with CRE (Morrill et al., 2015). The low level of resistance to carbapenems observed in the current study provides evidence that these antibiotics remain a viable option for the treatment of infections with ESBL-producing *Enterobacteriaceae* in this patient population. However, careful monitoring and judicious use of these last-line drugs is critical to ensure that they remain effective in treatment of potentially life-threatening multidrug-resistant infections.

#### Prediction of antimicrobial resistance between drug categories

Resistance to extended-spectrum cephalosporins was a significant predictor of resistance to penicillins, an expected finding given that β-lactamases mediating cephalosporin resistance also act upon penicillins (Ruppé, Woerther & Barbier, 2015). The other observed associations, however, may reflect co-transference of antimicrobial resistance genes *via* mobile genetic elements (MGEs). For example, extended-spectrum cephalosporin resistance was a significant predictor of resistance to fluoroquinolones and quinolones. Associations between plasmid-borne fluoroquinolone resistance and extended-spectrum β-lactamase production have previously been reported, consistent with these findings (Bouchakour et al., 2010; Crémet et al., 2011; Ruppé, Woerther & Barbier, 2015; Salah et al., 2019). For instance, the proportion of ciprofloxacin-resistant isolates among Gram negative bacteria isolated from hospital specimens in Tanzania was significantly higher among ESBL producers than non-ESBL producers (Mshana et al., 2009).

Aminoglycoside resistance was a significant predictor of resistance to carbapenems. Aminoglycoside resistance is frequently encountered among carbapenemase-producing *Enterobacteriaceae*, and can be mediated by aminoglycoside modifying enzymes acquired *via* MGEs (Livermore et al., 2011a; Livermore et al., 2011b; Ruppé, Woerther & Barbier, 2015). Carbapenemase- or ESBL-producing *Enterobacteriaceae* may carry resistance genes for multiple other drugs, limiting the available options for treatment (Ruppé, Woerther & Barbier, 2015). The observed relationships are consistent with previously reported mechanisms of antimicrobial resistance, and awareness of such associations may help inform evidence-based antimicrobial selection in situations where results of bacterial culture and sensitivity testing are not available.

#### Burden, distribution and determinants of MDR

The fact that approximately half of the *Enterobacteriaceae* isolates were MDR is quite concerning but is consistent with reports from recent studies conducted in Ethiopia that also reported high levels of MDR among enteric isolates (42.1% and 68.3%) (Teklu et al., 2019; Bitew & Tsige, 2020). Moreover, one of these studies also identified extensively-drug resistant (XDR) and pan-drug resistant (PDR) *Enterobacteriaceae* (Bitew & Tsige, 2020)*.* In contrast, MDR was much less common among isolates from clinical specimens collected from hospital patients in Uganda (27.8%) (Ampaire et al., 2015). Among *K. pneumoniae* isolated from invasive infections in a Kenyan hospital, as many as 79% were MDR, consistent with findings from the current study that 71% of *Klebsiella* spp. were MDR (Henson et al., 2017).

Compared to *E. coli*, *Proteus* spp. and *Salmonella* spp. had significantly lower odds of MDR. This is consistent with reports of differences in the prevalence of MDR across bacterial species from other studies (Teklu et al., 2019; Bitew & Tsige, 2020). The odds of MDR for *Enterobacteriaceae* isolated from male patients were significantly greater than that for female patients, which could reflect differences in previous antimicrobial exposure and/or gender differences in healthcare-seeking behavior. Such gender differences differ by both geographical location and sociodemographic factors, including income and educational attainment (Torres et al., 2019). A study conducted in northern Uganda reported that males were significantly more likely than females to self-medicate with antimicrobials, while a study from Sudan had contrasting results (Awad et al., 2005; Ocan et al., 2014). A review of factors influencing this practice in low- and middle-income countries (LMIC) suggests that it is generally more common among females, although this is not a consistent finding (Torres et al., 2019). A Nairobi survey reported that men are more likely to visit public health facilities over self-treatment, a finding that did not persist for private facilities, possibly due to gender disparities in resources (Muriithi, 2013). Thus, the association observed in the current study could reflect gender differences in self-medicating practices, care-seeking behavior, access to healthcare, education, or economic resources, among others. Further research is obviously warranted to explore this association.

### (b) *S. aureus*

*S. aureus* was the most frequently isolated Gram positive organism, mostly from bloodstream infections and wounds or abscesses, consistent with the findings of other studies (Omuse, Kabera & Revathi, 2015; Sangeda et al., 2017; Wangai et al., 2019). The reported prevalence of MRSA among *S. aureus* isolates has been quite variable across Africa (Kesah et al., 2003; Falagas et al., 2013), as well as between different facilities within Kenya (Omuse, Kabera & Revathi, 2015; Sangeda et al., 2017; Wangai et al., 2019; Iliya et al., 2020).

Consistent with findings of other studies in Kenya (Omuse, Kabera & Revathi, 2015; Sangeda et al., 2017; Wangai et al., 2019; Iliya et al., 2020), the majority (56.7%) of *S. aureus* isolates were resistant to folate pathway inhibitors. A somewhat lower but substantial proportion of *S. aureus* isolates (35%) were resistant to one or more tetracyclines, another commonly used class of antimicrobials. This is consistent with findings from other studies that reported levels ranging from 15.5% to 53.4% (Omuse, Kabera & Revathi, 2015; Sangeda et al., 2017; Wangai et al., 2019).

A much lower percentage of *S. aureus* isolates were MDR (16.7%) compared to *Enterobacteriaceae* (51%) in the current study. This is also much lower than findings from two other studies in the region, which reported that 63% and 64.8% of *S. aureus* isolates were MDR (Sangeda et al., 2017; Iliya et al., 2020). However, the criteria used to categorize isolates in the above studies as MDR were different from those used in the current study, limiting comparability. This calls for consistent use of a standardized classification scheme for identifying MDR isolates. This study used the framework recommended by the ECDC and CDC, which has the advantage of employing clinically and epidemiologically relevant categories of antimicrobials (Magiorakos et al., 2012).

### (c) *Pseudomonas* spp.

Resistance to aminoglycosides was by far the most common (42.1%) among *Pseudomonas* spp. isolates in the current study. This is much higher than findings of two Kenyan studies, where lower levels of resistance to amikacin (11%) and gentamicin (9 and 18%) were observed among *P. aeruginosa* isolates (Bejon et al., 2005; Wangai et al., 2019). However, other studies have reported findings similar to those of the current study. For example, resistance to amikacin and gentamicin were observed in at least 40% of *P. aeruginosa* isolates from rural and urban hospitals in Kenya (Mukaya et al., 2018; Thuo et al., 2019). Similar findings were reported by a study in Tanzania, where 34.8% of *Pseudomonas* spp. isolated from hospital patients exhibited resistance to gentamicin (Mshana et al., 2009). Although monotherapy with aminoglycosides is not recommended for treatment of suspected *P. aeruginosa* infections, these agents play an important role in combination therapy (Bassetti et al., 2018). The substantial proportion of aminoglycoside-resistant isolates observed in our study and others in the region should be taken into consideration by clinicians formulating a plan for empirical treatment.

The percentage of *Pseudomonas* spp. isolates resistant to antipseudomonal fluoroquinolones (15%) was considerably lower than to aminoglycosides, a finding that is consistent with those of some other studies (Mshana et al., 2009; Thuo et al., 2019). Fluoroquinolones, which have a broad spectrum of activity and the advantage of oral administration, may be recommended for treatment of certain suspected *P. aeruginosa* infections, either alone or as part of combination therapy (Bassetti et al., 2018). Reports of fluoroquinolone resistance also appear to vary markedly between different facilities. While a 2005 study found that just 2% of *P. aeruginosa* isolates from inpatients at a Kenya hospital were resistant to ciprofloxacin (Bejon et al., 2005), this was as high as 52.7% in another more recent hospital-based study (Mukaya et al., 2018). Given the high proportion of aminoglycoside-resistant isolates and the limited number of useful antimicrobials due to natural resistance mechanisms of *Pseudomonas,* it is important to carefully monitor resistance to these agents (Bassetti et al., 2018)*.*

### Strengths and limitations

Firth logit models used in this study generate better estimates than ordinary logistic models, which use maximum likelihood estimation which suffer from small-sample bias (Heinze & Schemper, 2002). Parameter estimates of Firth models are generated using penalized likelihood, which reduces the small-sample bias associated with maximum likelihood estimation (Firth, 1993). Additionally, penalized likelihood estimations produce finite, consistent estimates of regression parameters in situations when maximum likelihood estimates do not exist due to complete or quasi-complete separation (Heinze, 2006; Williams, 2019). Therefore, although sample size was a statistical challenge, it was adequately addressed using this modeling approach.

Since this was a retrospective study that used secondary data, the investigators did not determine the antimicrobial agents included in the panel for susceptibility testing. The panels used by the diagnostic laboratory were not consistent for all isolates of the same bacterial species, highlighting the need for standardization of laboratory procedures and reporting in the region. Detailed patient medical history was not available. Therefore, the associations of AMR or MDR with clinical factors (such as current and prior antimicrobial use, underlying health conditions, primary diagnosis, and treatment outcomes) could not be investigated. Information about whether the isolated organisms were considered primary infectious agents was also not available. For instance, it is possible that some isolates from stool specimens were commensal organisms and may not have been responsible for clinical signs. However, commensal bacteria represent a potential reservoir for antimicrobial resistance genes that may be transferred to pathogens (Singh, Verma & Taneja, 2019). Therefore, monitoring patterns of AMR in these organisms provides valuable information.

Despite the above limitations, the findings of this study provide useful information for clinicians and contribute to literature on AMR patterns in Kenya specifically and sub-Saharan Africa in general. Moreover, the study focused on clinically and epidemiologically relevant antimicrobial categories as well as pathogens of public health importance. The study findings are therefore useful to guide future AMR surveillance programs and directions for future research.

## Conclusions

The high levels of extended-spectrum cephalosporin resistance and MDR among *Enterobacteriaceae* observed in this study are concerning. However, the relatively low levels of MRSA and MDR *S. aureus* and an absence of carbapenem resistance among *Pseudomonas* spp. isolates is encouraging. This may suggest that last-line drugs remain available for the treatment of infections with resistant *S. aureus* and *Pseudomonas* spp. isolates when necessary. However, continued antimicrobial stewardship, adherence to biosecurity practices, and surveillance are warranted to ensure this does not change.

## Supplemental Information

10.7717/peerj.11958/supp-1 Supplemental Information 1 Raw data used in the study

## Additional Information and Declarations

### Data Availability

The following information was supplied regarding data availability: The study data are available in the Supplemental File.
